# Sohlh2 promotes pulmonary fibrosis via repression of p62/Keap1/Nrf2 mediated anti-oxidative signaling pathway

**DOI:** 10.1038/s41419-023-06179-z

**Published:** 2023-10-24

**Authors:** Lanlan Liu, Xiaoli Zhang, Ruihong Zhang, Liyan Wang, Sujuan Zhi, Xiaoning Feng, Xuyue Liu, Ying Shen, Jing Hao

**Affiliations:** 1 Key Laboratory of the Ministry of Education for Experimental Teratology, Department of Histology and Embryology, School of Basic Medical Sciences, Cheeloo College of Medicine, Shandong University, 44 Wenhua Xi Road, Jinan, Shandong 250012 P. R. China; 2 Morphological Experimental Center, School of Basic Medical Sciences, Cheeloo College of Medicine, Shandong University, 44 Wenhua Xi Road, Jinan, Shandong 250012 P. R. China

**Keywords:** Respiration, Respiratory tract diseases, Apoptosis, Stress signalling

## Abstract

Disturbance in the redox balance of alveolar epithelial cells (AECs) was considered as a causal factor for pulmonary fibrosis. The regulatory mechanisms of redox hemostasis in the development of pulmonary fibrosis remain largely unknown. Using a type II AEC-specific Sohlh2 conditional knock-in (CKI) mouse model, we found that Sohlh2, a basic HLH transcription factor, accelerated age-related pulmonary fibrosis. High-fat diet (HFD) resulted in a tremendous increase in lung inflammation and fibrotic changes in the lung tissues of Sohlh2 CKI mice. Sohlh2 overexpression led to a significant rise of intracellular ROS and apoptosis in the lung, mouse primary AECIIs, and human A549 cells, which was attenuated by ROS inhibitor (NAC). Sohlh2 enhanced oxidative stress via repressing p62/Keap1/Nrf2 mediated anti-oxidative signaling pathway. p62, a direct target of Sohlh2, mediated Sohlh2 effects on ROS generation and apoptosis in A549 cells. Hence, our findings elucidate a pivotal mechanism underlying oxidative stress-induced pulmonary fibrosis, providing a framework for aging-related disorder interventions.

## Introduction

Idiopathic pulmonary fibrosis (IPF) is a chronic, progressive lung interstitial disease with a median survival of 3 ~ 5 years [1]. The pathophysiology of IPF is characterized by the aberrant accumulation of fibrotic tissue and destruction of the alveolar structure in the lung, which frequently occurs in the elderly male population [2]. Currently, patients with IPF have very limited treatment options. Exploring the molecular mechanism of IPF pathogenesis is expected to provide a novel target for IPF therapy.

Emerging evidence has demonstrated that an imbalance in the redox status of alveolar epithelial cells was considered as a causal factor for IPF [3]. The patients with IPF and animal models of pulmonary fibrosis show significantly increased ROS production, cell injury, and apoptosis in lung epithelial cells [4]. Despite covering ~5% surface lining of the lung, type II alveolar epithelial cells (AECIIs) serve as a reservoir of cells that proliferate and differentiate into type I alveolar epithelial cells (AECIs) in response to pulmonary injury [5, 6]. Dysfunctional mitochondria and ROS accumulation are evident in AECIIs in the lungs of patients with IPF [7, 8]. Apoptosis of AECs has been shown to initiate fibrosis, and secreted factors from senescent epithelial cells can induce myofibroblast differentiation in vitro [9, 10]. However, the precise molecular mechanisms of redox imbalance in AECIIs during the development of IPF remain largely unknown.

p62/Keap1/Nrf2 signaling pathway plays a crucial role to maintain redox hemostasis. SQSTM1/p62, a selective autophagy adapter, recruits Kelch-like ECH-associated protein 1 (Keap1) for degradation by autophagosomes and release of Nuclear factor erythroid 2-related factor 2 (Nrf2) to the nucleus. Nrf2 induces the expression of antioxidant genes including glutathione S-transferase (GST), NAD(P)H: quinone oxidoreductase 1 (NQO1), Hemeoxygenase1 (HO1), and ferritin heavy chain 1 (FTH1), which have been shown to protect against pulmonary fibrosis in murine models [11–13]. AECs isolated from Nrf2-deficient mice are prone to oxidant-induced cell death and impaired proliferation [14]. Therefore, it is significant to reveal novel regulators in p62/Keap1/Nrf2 signaling pathway.

Spermatogenesis and oogenesis specific bhlh transcription factor 2 (Sohlh2) belongs to the superfamily of basic helix-loop-helix (bhlh) transcription factors [15]. Sohlh2 knockout in mice results in the blockade of the development of spermatogonia and primordial follicles [16]. In human tissues, Sohlh2 distributes widely, especially in epithelial cells [17]. Our results have confirmed that Sohlh2 is a novel tumor suppressor through its regulation of cell cycle mediators, such as p21 and cyclin D1 [18]. Recently, we found that Sohlh2 was involved in the regulation of oxidative stress caused by chemotherapeutic agents in cancer cells (data not shown). The regulatory mechanisms of Sohlh2 in the development of IPF are required to explore.

In the present study, the mechanisms associated with how Sohlh2 induced age-related or HFD-induced redox imbalance, and accelerated lung fibrosis, were investigated at a multi-system level. Using a mouse model of Sohlh2 CKI in AECIIs, cultured mouse primary AECIIs, and A549 cells, we demonstrated that Sohlh2 attenuated the activation of the p62/Keap1/Nrf2 signaling pathway to trigger ROS production. Moreover, we found that p62 was a direct target gene of Sohlh2, and mediated the effects of Sohlh2 on oxidative stress damage and lung fibrosis. Our findings provided evidence that Sohlh2 may serve as a potential therapeutic target in IPF.

## Materials and methods

### Generation of Sohlh2 CKI mice

*Sohlh2*^loxP/loxP^ and *Sftpc*^CreERT2+^ mice were both purchased from Cyagen (Suzhou, China). To generate mice with tamoxifen-inducible Sohlh2 expression specifically in AECIIs, *Sohlh2*^loxP/loxP^ were crossed to *Sftpc*^CreERT2+^ mice. To induce recombination by CreERT2, 5 consecutive intraperitoneal tamoxifen (100 mg/kg/dose; Sigma-Aldrich, MO, USA) injections, were given from 6 weeks of age. *Sohlh2*^loxP/loxP^ *Sftpc*^CreERT2-^ mice were used as the control for experiments. All mice were kept in a controlled environment with 24 ~ 26 °C, 50% ~ 60% humidity, and 12 h cycle of night and day. DNA samples were extracted from lungs obtained from *Sohlh2*^loxP/loxP^*Sftpc*^CreERT2-^ (Control) and *Sohlh2*^loxP/loxP^*Sftpc*^CreERT2+^ (Sohlh2 KI) mice using DNeasy Blood and Tissue Kit (Qiagen, Germany), which were used for genotyping through PCR reactions and the subsequent resolution by agarose gel electrophoresis. The primer sequences are shown in Extended Data Table 1. All animal experiments and procedures in this study were approved by the Committee on Ethical Use of Animals of the School of Basic Medicine of Shandong University and were performed in compliance with all relevant ethical regulations.

### HFD-induced murine lung fibrotic model

Briefly, 8-week-old mice were fed with HFD (Medicinece, Jiangsu, China) for 8 weeks and sacrificed for subsequent analysis. In parallel experiments, Mice were randomly divided into the Control/HFD, Sohlh2 KI/HFD, Control/HFD + NAC, and Sohlh2 KI/HFD + NAC groups. Each group contained 5 mice. The phenotype was analyzed by a blind investigator. Four-group mice were fed with HFD for 8 weeks, at the same time, the mice in Control/HFD + NAC and Sohlh2 KI/HFD + NAC groups were fed with 100 μL NAC solution at the concentration of 30 mg/mL every day for 8 weeks.

### Bronchoalveolar lavage fluid (BALF) collection and cell count

Briefly, after the mice were euthanized, the lungs were lavaged with 0.8 mL ice-cold PBS for three times, and the BALF was collected. After centrifugation for 5 min at 4 °C, the supernatant was stored at −80 °C for subsequent experiments. The sedimented cell pellets were re-suspended in 100 μL PBS. The cell number was quantified by a hemocytometer, and cytospin slides were prepared using 40 μL of the cell suspension with 160 μL of PBS. Slides were stained using Wright-Giemsa staining, and the numbers of macrophages and neutrophils were counted in a total of at least 200 cells.

### Isolation of murine AECIIs

The Control and Sohlh2 KI mice were euthanized by intraperitoneal injection of 1% pentobarbital, and a thoracotomy was performed. The lungs were excised and were digested in 2 mL 1 g/L trypsin (including DnaseI 0.01 g/L). After filtration sequentially through a 200-mesh Nylon screen and centrifugation, the supernatant was discarded and the cell pellets were suspended in DMEM.

The lung cell suspension was inoculated into the petri dish coated with mouse IgG and incubated for 40 min in a 5%CO_2_ cell incubator at 37°C. The liquid containing unadhered cells was sucked out and inoculated in another petri dish coated with mouse IgG and incubated for 40 min at 37°C for two times. Finally, the unadhered cells were sucked out and centrifugation, the supernatant was discarded and the cell pellets were suspended in DMEM, then cultured in a 5% CO_2_ cell incubator at 37°C. After 24 h, ~90% of adherent cells were AECIIs. The morphological characteristics and growth of cells were observed by an inverted microscope every day.

### Immunofluorescent staining of isolated AECIIs

Cells were fixed in a 4% fixative solution and permeabilized with 0.2% Triton X-100. After blocking, the cells were incubated with primary antibodies against SP-C (Affinity, USA), and then a fluorescent secondary antibody. Nuclei were stained with DAPI (Beyotime, Haimen, China). Images were obtained under a fluorescence microscope (Olympus, Tokyo, Japan) using CellSens Dimension software.

### Flow cytometry analysis

For apoptosis analysis, cells were analyzed with Annexin V-FITC/PI Apoptosis Detection Kit (Vazyme, Nanjing, China). For analysis of ROS, mouse primary AECIIs and human A549 cells were untreated or treated with 300 µM PA for 24 h, then collected and suspended in 1 mL PBS containing 2 µM DHE (Beyotime, Haimen, China) in the dark for 30 min at 37 °C. After incubation, samples were examined by flow cytometry (CytoFLEX, Beckman Coulter, CA, USA), and the data were analyzed using CytExpert software (Beckman Coulter, CA, USA).

### Real-time qPCR

The extraction of total RNA, generation of cDNA, and RT-qPCR were achieved as described in our previous study [17]. Gene expression was measured by 2^-ΔCt^, and the relative gene expression was assayed by 2^-ΔΔCt^. Primers used in this study were synthesized by the Beijing Genomics institution (Beijing, China), and the primer sequences are shown in Extended Data Table 2.

### Western blot analysis

Total proteins from the lung tissues or cells were extracted and analyzed using Western blot as described in our previous study [19]. Frozen lung tissues were homogenized and lysed in RIPA buffer (Beyotime, Haimen, China) containing a protease inhibitor (Solarbio, Beijing, China). Nuclear and cytosol extracts were prepared using the Nuclear Protein Extraction Kit (Solarbio, Beijing, China), according to the manufacturer’s instructions. Subsequently, these cellular fractions underwent Western blot assays. Histone H3 was used as a nuclear fraction marker, and β-Tubulin was used as a loading control for cytosol protein [20]. The protein concentrations were measured with BCA Protein Quantification Kit (Vazyme, Nanjing, China). Samples mixed with the loading buffer were separated on a 10% SDS-PAGE gel. After transferring to polyvinylidene fluoride membranes (Millipore, Bedford, MA) by electrotransfer, the membranes were blocked with 5% non-fat milk at room temperature for 2 h. The membranes were probed with appropriate primary antibody at 4 °C overnight, followed by incubation with peroxidase-conjugated anti-rabbit (or anti-mouse) IgG antibody for 2 h at room temperature. The interaction was monitored with a BeyoECL Star (Beyotime, Haimen, China). The antibodies used in the study are shown in Extended Data Table 3.

### Cell culture and treatment

The human cell line A549 was purchased from the Cell Bank of Type Culture Collection of the Chinese Academy of Sciences (Shanghai, China), and was maintained in RPMI 1640 medium containing 10% FBS and 1% penicillin-streptomycin. The cell lines were authenticated by short tandem repeat (STR) profiling and tested free of mycoplasma. For the stable overexpression or knockdown of Sohlh2 in A549 cells, DNA transfection was performed as previously described [21]. Briefly, A549 cells were seeded at 25% confluence in 10-cm plates the day before transfection. Two days later, infected cells were selected with 1 μg/mL puromycin (Sigma-Aldrich, MO, USA) for 2 weeks. The human A549 cells and mouse primary AECIIs were treated with 300μΜ PA, and the corresponding control group was treated with PBS. The cells were seeded on coverslips, which were pre-placed in 24-well plates. After the cell adheres to the coverslips, the cells were exposed to 300μΜ PA for 48 h after pretreatment with or without 5 mM NAC.

### MDA assay

The up-right lung lobes of mice were homogenized in PBS at a ratio of 1:10 (weight: volume). A549 cells were collected and homogenized. The levels of malondialdehyde (MDA) in the lungs, serum, and A549 cell homogenate were assessed by corresponding kits following the manufacturer’s instructions (Jiancheng Bioengineering Institute, Nanjing, China).

### ELISA assay

The contents of tumor necrosis factor-alpha (TNF-α), transforming growth factor-beta1 (TGF-β1), and interleukin-6 (IL-6) in BALF, cell culture supernatant, and the lung tissues were measured using ELISA kits (Excell Bio, Jiangsu, China). The contents were assayed by comparison of the optical density (450 nm and 570 nm) with the standard curve.

### Histological analysis

After fixation with 4% paraformaldehyde (PFA) and paraffin embedding, lung sections (4μm) were used in hematoxylin and eosin (H&E), Masson’s trichrome, immunofluorescence staining assays. For the immunofluorescence assay, 5% bovine serum albumin (BSA) was used to block the sections, after which the tissues were incubated with anti-Sohlh2, anti-α-SMA, and anti-FN overnight at 4 °C. Then, a fluorescent secondary antibody was added for 1 h at 37 °C. The sections were covered with an antifade solution containing DAPI (Beyotime, Haimen, China). The fluorescence signals of the lung tissues were visualized with fluorescence microscopy (Olympus, Tokyo, Japan) using CellSens Dimension software. The antibodies used for immunofluorescence are shown in Extended Data Table 3.

### TUNEL staining

TUNEL staining for the analysis of apoptosis was performed using One-step TUNEL In Situ Apoptosis Kit (Elabscience Biotechnology, TX, USA). Images were obtained under a fluorescence microscope (Olympus, Tokyo, Japan) using CellSens Dimension software. TUNEL-positive cells displayed green staining within the nucleus, and the number of TUNEL-positive cells was counted in three nonoverlapping microscopic fields by a blinded person under high power magnification and displayed as a percentage.

### DHE staining

Intracellular ROS levels were quantitatively analyzed with dihydroethidium (DHE) (Beyotime, Haimen, China) by measuring fluorescent intensity. The cells were seeded on coverslips. The freshly prepared DHE (2 μM) was incubated with the cells in the dark for 30 min at 37 °C. The fluorescent intensity was proportional to ROS levels, and images were captured with a fluorescence microscope (Olympus, Tokyo, Japan) using CellSens Dimension software.

After routine dewaxing and hydration of paraffin sections of mouse lung tissues, the sections were incubated with freshly prepared DHE (2 μM) in the dark for 30 min at 37 °C. After washing three times with PBS, a coverslip was placed on the section, and fluorescent images were captured using a fluorescence microscope (Olympus, Tokyo, Japan).

### Transmission Electron Microscopy (TEM)

Fixatives for TEM sample preparation were composed of 4% paraformaldehyde, 2.5% glutaraldehyde, and 0.02% picric acid in 0.1 M sodium cacodylate buffer. Murine lungs were inflated with 1.2 mL TEM fixative and were then excised and transferred to a 50 mL polypropylene tube containing 10 mL TEM fixative. A TEM (FEI, OR, USA) was used to obtain images with an accelerating voltage of 200 kV. AECIIs were identified according to the appearance of lamellar bodies and the microvilli at the apical cell membrane.

### ChIP assay

ChIP analysis was performed following a previously described protocol [22]. A549 cells stably expressing Sohlh2 or control vector were prepared using a Simple ChIP Enzymatic Chromatin IP Kit (Cell Signaling Technology, MA, USA) following the manufacturer’s guidelines. A549 cells were treated cross-linked with 37% formaldehyde at a final concentration of 1% at room temperature for 10 min. Fragmented chromatin was treated with nuclease and subjected to sonication. Chromatin immunoprecipitation was performed with rabbit anti-Sohlh2 antibody (Novus, CO, USA) and normal rabbit IgG. After reverse cross-linking and DNA purification, immunoprecipitated DNA was quantified by real-time PCR using UltraSYBR Mixture (CWBIO, Jiangsu, China) with primers for Sohlh2 binding sites in the p62 promoter. The human p62 promoter-specific primers used were as follows: forward 5’-GAAGCTCGGGGTGCGG-3’, reverse 5’-CGGTCCTGGGACTCCCTT-3′. Primers as negative control sites were 5’-TTTCGGAAGCGTTTTCCC-3’ and 5’-AGCGCGTTCATTCAGGAA-3’. Fold enrichment was calculated based on the threshold cycle (CT) value of the IgG control using the comparative CT method.

### Luciferase reporter assay

A549 cells were cultured in a 24-well plate and transiently transfected with Sohlh2 overexpression plasmid, p62 promoter firefly luciferase reporter construct, and Renilla luciferase plasmid (Promega, WI, USA) using Lipofectamine2000 (Invitrogen, CA, USA). Luciferase activities were determined 48 h after transfection using a dual-luciferase reporter assay system (Vazyme, Nanjing, China). Results were represented as the ratio of Firefly to Renilla luciferase activity and normalized to vector control.

### Statistical analysis

All experiments were independently repeated three times. Results were shown as the mean ± SD values. All data were analyzed with GraphPad Prism 7 software (San Diego, CA, USA). The Student *t*-test was used to assess the significant difference between groups. In these analyses, *P*-value < 0.05 was considered as a statistically significant difference.

## Results

### The generation of inducible AECII-specific Sohlh2 knock-in mice

**Fig. 1:**
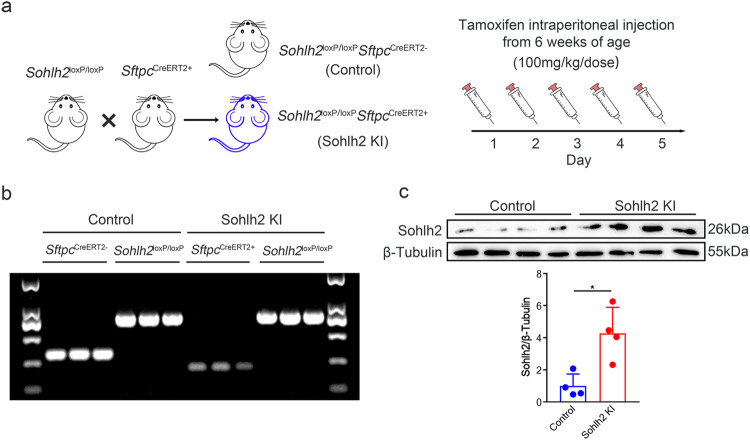
The generation of inducible AECII-specific Sohlh2 knock-in mice. **a** Schema demonstrating the process of AECII-specific mice (Sohlh2 KI) mice generation using tamoxifen-inducible *Sftpc*-promoter driven CreERT2. *Sohlh2*^loxP/loxP^*Sftpc*^CreERT2-^ male mice were used as controls. **b** *Sohlh2*^loxP/loxP^ mice were crossed to *Sftpc*^CreERT2+^ mice, and genotypes of offspring mice were identified by PCR (*n* = 3 mice per group). **c** Representative images and quantification analysis of Western blot assay showing Sohlh2 protein levels in the Control and Sohlh2 KI mouse lungs (*n* = 4 mice per group). Data are presented as the mean ± SD. *P < 0.05.

To elucidate the function of Sohlh2 in AECIIs, conditionally knock-in Sohlh2 genes in murine AECIIs were generated by the Cre-loxP system. Genetically modified mice harboring Sohlh2 flanked by loxP-stop-loxP sites were crossed with *Sftpc*^CreERT2+^ mice (Fig. 1a). Tamoxifen treatment resulted in the selective expression of Sohlh2 genes in AECIIs (*Sohlh2*^loxP/loxP^*Sftpc*^CreERT2+^, Sohlh2 KI mice) as confirmed by genotyping (Fig. 1b) and immunoblotting (Fig. 1c). *Sohlh2*^loxP/loxP^*Sftpc*^CreERT2-^ mice were used as controls. These results showed that the AECII-specific Sohlh2 knock-in murine model was successfully constructed.

### Sohlh2 overexpression in AECIIs causes spontaneous age-related pulmonary fibrosis

**Fig. 2:**
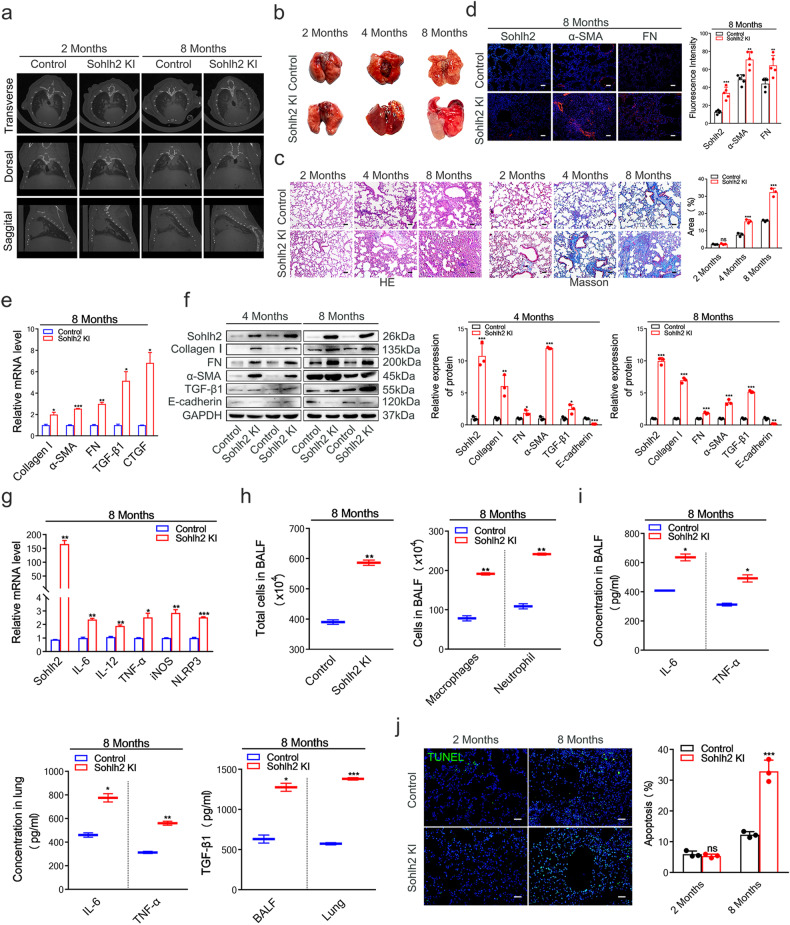
Sohlh2 overexpression in AECIIs causes spontaneous age-related pulmonary fibrosis. **a** Representative images of reconstructed anatomical X-ray MicroCT obtained from 2 M and 8 M mice showed parenchymal opacity. **b** Representative images showing gross lung morphology of 2-, 4-, and 8-month-old mice. **c** Representative images and quantification analysis of HE and Masson staining in sections from the indicated 2 M, 4 M, and 8 M lungs of the Control and Sohlh2 KI mice. Scale bars: 50 μm. **d** Representative images of immunofluorescence staining in the lung tissue samples of 8 M mice showing Sohlh2 (red), α-SMA (red), FN (red), and nuclei (blue). Scale bars: 50 μm. **e**, **f** qPCR and Western blot analysis of the expression of fibrosis-related genes in the indicated Control- and Sohlh2 KI-lungs. **g** qPCR analysis of proinflammatory cytokines in the Control- and Sohlh2 KI-lungs. **h** The total cells, macrophages, and neutrophils in the BALF of 8 M Control and Sohlh2 KI mice. **i** ELISA analysis of proinflammatory cytokines in the indicated lungs and BALF. **j** Representative images and quantification analysis of TUNEL staining in sections from the indicated 8 M Control- and Sohlh2 KI-lungs. Scale bars: 50 μm. Data are presented as the mean ± SD. ns. *P* > 0.05, *P < 0.05, **P < 0.01, and ***P < 0.001.

To clarify the role of Sohlh2 overexpression in AECIIs in the occurrence and progression of pulmonary fibrosis. We evaluated fibrotic responses in the lungs of 2-, 4-, 8-month-old Sohlh2 KI male mice and their littermate controls. MicroCT analysis of 8-month-old Sohlh2 KI mice showed an obvious change in the lung density and increased parenchymal opacity compared to the Control mice, and at the age of 2 months, Sohlh2 KI mice were indistinguishable from the controls (Fig. 2a). Morphologically, the lung structure of 2 M Control and Sohlh2 KI mice was normal, and there was no significant difference in lung morphology between the two groups, while Sohlh2 KI mice showed age-related lung collapse and fibrogenesis (Fig. 2b). There was an increment in the thickness of the alveolar wall, interstitial infiltrated inflammatory cells, and the collapse of the alveoli in the lungs of mice as they aged, which were evidenced by the histological evaluation (Fig. 2c). Sohlh2 KI mice developed age-related progressive lung fibrosis, Masson’s trichrome staining showed accelerated collagen deposition in the Sohlh2 KI group, while there were indistinguishable between the two groups at the age of 2 months (Fig. 2c). The severe fibrosis was accompanied by upregulated expression of fibrotic genes, including Collagen I, fibronectin (FN), α-SMA, TGF-β1, CTGF, and E-cadherin (Fig. 2d–f). These results indicate that Sohlh2 overexpressing in AECIIs promotes age-related pulmonary fibrosis.

To determine the inflammatory injury of lung tissues in 8-month-old Sohlh2 KI mice and the control group, we analyzed the inflammatory responses and cell apoptosis in lung tissues. The data showed the expression of proinflammatory cytokines was upregulated in the Sohlh2 KI mice (Fig. 2g). Similarly, the number of total cells, macrophages, and neutrophils dramatically aggrandized in the BALF of 8-month-old Sohlh2 KI mice compared to the Control mice (Fig. 2h). The result of the ELISA assay showed that Sohlh2 overexpression in AECIIs led to augmenting the secretion of IL-6, TNF-α, and TGF-β1 in the lungs and BALF of 8-month-old Sohlh2 KI mice (Fig. 2i). To determine the cell apoptosis in lung tissues, a TUNEL assay was performed in the lung sections of 2- and 8-month-old Sohlh2 KI and their controls. The numbers of TUNEL-positive cells increased in 8-month-old Sohlh2 KI lungs compared to the Control mice, while at the age of 2 months, there were no significant changes between the two groups (Fig. 2j). To sum up, Sohlh2 overexpression in AECIIs enhanced inflammatory responses and cell apoptosis in the mouse lung tissues.

### Sohlh2 aggravates the progression of pulmonary fibrosis induced by HFD

**Fig. 3:**
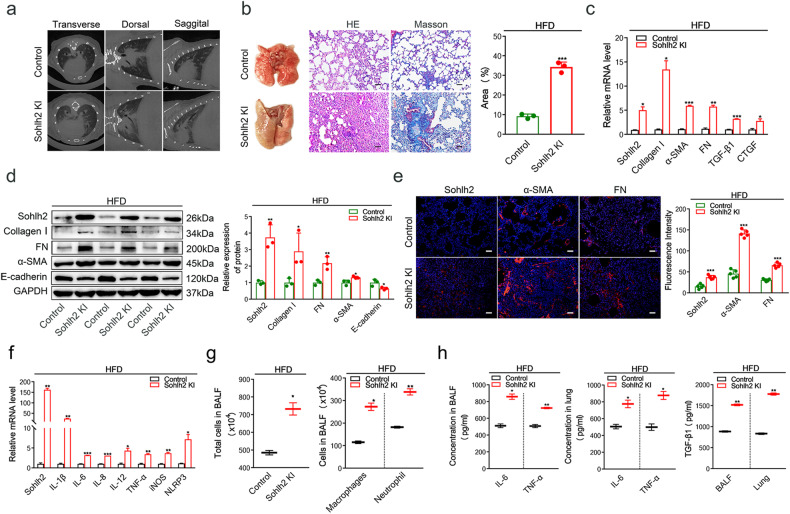
Sohlh2 aggravates the occurrence and progression of pulmonary fibrosis induced by HFD. **a** Representative images of reconstructed anatomical X-ray MicroCT showing parenchymal opacity of lungs obtained from HFD-fed mice. **b** Representative images showing gross lung morphology of HFD-fed mice (left), *n* = 5 mice per group; representative photomicrographs of HE-stained lung sections (middle). Scale bars: 100 μm; representative images of Masson’s trichrome-stained lung sections at 8 weeks after HFD treatment (right). Scale bars: 100 μm. The histogram on the right is the statistical analysis of Masson’s trichome staining. **c**, **d** qPCR and Western blot analysis of profibrotic gene expression at mRNA and protein levels from the indicated lung tissues of HFD mice. **e** Immunofluorescence staining analysis of representative mice lung tissue samples of HFD mice showing Sohlh2 (red), α-SMA (red), FN (red), and nuclei (blue). Scale bars: 50 μm. **f** qPCR analysis of proinflammatory cytokines mRNA levels from the indicated lung tissues after 8-week HFD treatment. (*n* = 3). **g** The total cells, macrophages, and neutrophils in the BALF of the Control and Sohlh2 KI mice after 8-week HFD treatment. **h** ELISA analysis of proinflammatory cytokines in the Control- and Sohlh2 KI-lungs and the indicated BALF after 8-week HFD treatment. Data are presented as the mean ± SD. *P < 0.05, **P < 0.01, and ***P < 0.001.

Metabolic stress could cause pulmonary fibrosis. To explore the role of Sohlh2 in pulmonary fibrosis induced by metabolic stress, we fed 8-week-old Sohlh2 KI mice with HFD containing 60% fat by kilocalories for 8 weeks and assessed their lung morphology, inflammatory responses, and collagen deposition after HFD treatment. HFD-fed Control and Sohlh2 KI mice gained a similar amount of body weight. MicroCT analysis of HFD-fed Sohlh2 KI mice showed a marked change in the lung density and heighten parenchymal opacity compared with the Control mice (Fig. 3a). Morphologically, HFD-fed Sohlh2 KI mice had a more severe lung collapse and more fibrous nodules compared to the Control mice (Fig. 3b). After HFD treatment, Sohlh2 enhanced lung inflammatory responses as well as destructed lung architecture with thickened alveolar septa and collapsed alveolar spaces, which were evidenced by the histological evaluation (Fig. 3b). Masson trichrome-stained sections showed more severe fibrosis in the lungs of HFD-fed Sohlh2 KI mice compared with the Control mice (Fig. 3b). Sohlh2 elevated the expression of TGF-β1, as well as other genes (Collagen I, FN, α-SMA, CTGF, and E-cadherin) involved in lung fibrogenesis after HFD treatment (Fig. 3c, d). Similarly, HFD-induced Sohlh2 KI lungs are characterized by architectural tissue remodeling with the accumulation of FN and α-SMA (Fig. 3e). To determine the molecular consequences of HFD feeding, we performed qPCR analysis and observed considerably upregulated expressions of genes involved in proinflammatory cytokines (IL-1β, IL-6, IL-8, and TNF-α) in the lungs of the Sohlh2 KI mice (Fig. 3f). Moreover, Sohlh2 markedly enlarged the number of total cells, macrophages, and neutrophils in BALF (Fig. 3g), and significantly improved the abundance of IL-6, TNF-α, TGF-β1 in BALF and lung tissues of HFD-fed mice (Fig. 3h). The analyses above clearly elucidate that Sohlh2 aggravates pulmonary fibrosis induced by HFD.

### Sohlh2 enhances oxidative stress in the lungs and cultured AECIIs

**Fig. 4:**
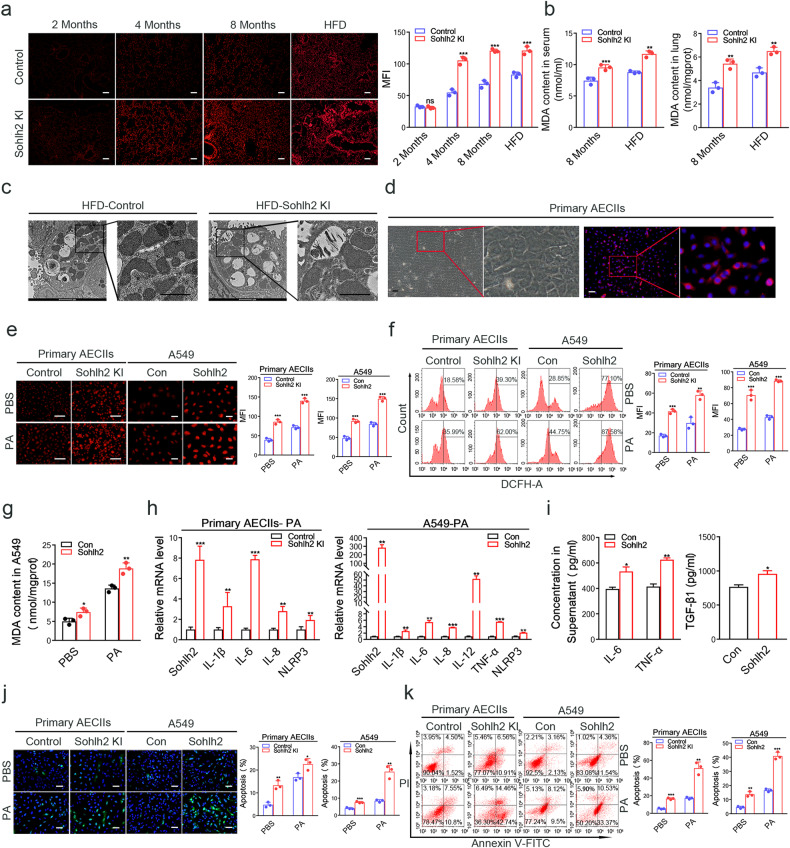
Sohlh2 enhances oxidative stress in the lungs and cultured AECIIs. **a** Representative photographs of DHE fluorescent imaging of lung tissue sections. Scale bars: 100 μm. **b** The levels of MDA in the lung tissues of 8 M and HFD mice were detected. **c** Representative TEM images of AECIIs from the Control and Sohlh2 KI mice after 8-week HFD treatment. Mitochondrial profiles showed enlarged swollen mitochondria in Sohlh2 KI AECIIs. Boxed regions are enlarged at the right. Scale bars: 1 μm. **d** Under an inverted phase-contrast microscope, the morphology of murine primary AECIIs (left). Scale bars: 50 μm; the expression of cell surfactant protein SP-C was measured by immunofluorescence staining (right). Scale bars: 50 μm. **e** ROS levels were measured by DHE fluorescent intensity in murine primary AECIIs and A549 cells treated with or without 300 μM PA. Scale bars: 50 μm. **f** Detection of ROS level and average immunofluorescence intensity of mouse primary AECIIs and human A549 cells treated with or without PA by FACS. **g** The levels of MDA were shown in Sohlh2 overexpression A549 cells treated by PA. **h** qPCR analysis of proinflammatory cytokines mRNA levels from mouse primary AECIIs and human A549 cells treated by PA. (*n* = 3). **i** ELISA analysis of IL-6, TNF-α, and TGF-β1 in the culture medium of Sohlh2 overexpression A549 cells treated by PA. **j** Representative images and quantification analysis of TUNEL staining in mouse primary AECIIs and human A549 cells treated with or without PA. (*n* = 3). Scale bars: 20 μm. **k** Percentages of Annexin V-positive cells upon PA treatment were examined by FACS. Cells treated with PBS served as the control. (*n* = 3). Data are presented as the mean ± SD. ns. *P* > 0.05, *P < 0.05, **P < 0.01, and ***P < 0.001.

Excessive ROS production causes oxidative stress, leading to cell damage. Numerous studies have reported that oxidative stress is involved in the pathogenesis of pulmonary fibrosis [23, 24]. DHE staining analysis in the lung tissues of 2-, 4-, 8-month-old mice and HFD-fed mice showed that ROS levels in lung tissues increased gradually with age, and Sohlh2 significantly augmented the production of ROS in lung tissues. Following HFD stimulation, this effect was more obvious (Fig. 4a). One of the features of oxidative stress is the high level of MDA, a product of lipid peroxide. The results showed that the MDA content in Sohlh2 KI lung tissue homogenate and serum of 8-month-old and HFD-fed mice was significantly higher than that of the Control mice (Fig. 4b). AECs are the primary site of injury and trigger the fibrotic responses [25]. We next examined mitochondrial ultrastructural changes in AECIIs in the murine model of HFD-induced lung fibrosis through TEM. AECIIs of Sohlh2 KI mice exposed to HFD showed swollen mitochondria with disrupted cristae compared to the controls (Fig. 4c). These results demonstrate that Sohlh2 could promote the occurrence and progression of pulmonary fibrosis by aggravating oxidative stress and mitochondrial damage of AECIIs in mouse lung tissues.

The accumulation of ROS in AECIIs is the main reason for the alveolar epithelial damage [26, 27]. We isolated AECIIs from Sohlh2 KI lungs and the control group. As shown in Fig. 4d, mouse primary AECIIs were round or cuboidal, with obvious fine particles in the cytoplasm, obvious nuclei, and island-shaped growth. We detected SP-C expression by immunofluorescence staining, a surface-active protein specifically expressed in AECIIs. The results showed that about 90% of the cultured cells expressed SP-C protein (red) (Fig. 4d), indicating that we successfully obtained mouse primary AECIIs. To mimic the metabolic stress in vivo, we examined the oxidative stress damage of mouse primary AECIIs and human A549 cells treated with 300 μM PA. The data showed that Sohlh2 significantly strengthened DHE fluorescent intensities and the levels of MDA in AECIIs (Fig. 4e–g), while Sohlh2 knockdown reduced MDA levels and DHE fluorescent intensities in AECIIs (Extended Data Fig. 1a–c). These analyses indicate that Sohlh2 enhances oxidative stress in mouse and human AECIIs.

Excessive ROS could lead to cell apoptosis and secretion of the proinflammatory cytokines[4, 25, 28]. We detected the expression level of proinflammatory cytokines and apoptosis in AECIIs treatment with PA for 48 h. The results of qPCR and ELISA assays showed that Sohlh2 significantly upregulated the expression of proinflammatory cytokines (IL-1β, IL-6, IL-8, and TNF-α) in mouse primary AECIIs and human A549 cells (Fig. 4h, i), while knockdown Sohlh2 resulted in the opposite effects (Extended Data Fig. 1d, e). We further detected the cell apoptosis of PA-induced AECIIs by TUNEL staining and FACS analysis. The results showed that Sohlh2 promoted the percentage of apoptotic cells in mouse primary AECIIs and A549 cells (Fig. 4j, k), while the percentage of cell apoptotic cells was significantly lower in the Sohlh2 knockdown group compared to the control group (Extended Data Fig. 1f, g). Collectively, the data suggest that Sohlh2 enhances oxidative stress damage in AECIIs treatment with PA.

### NAC attenuated Sohlh2-mediated oxidative stress in AECIIs and pulmonary fibrosis

**Fig. 5:**
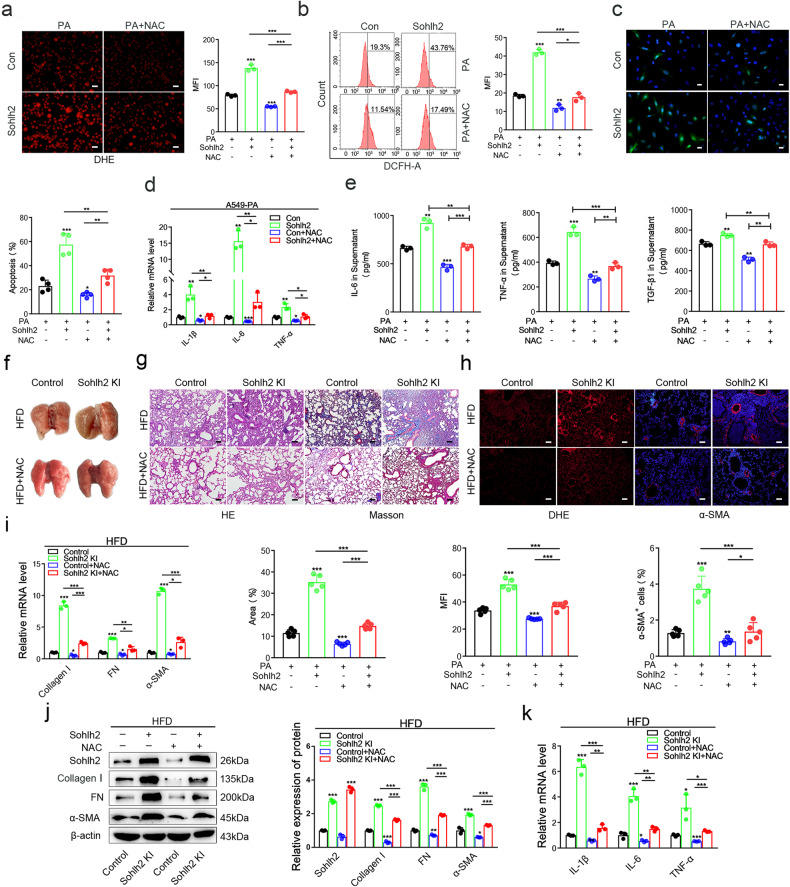
NAC attenuated Sohlh2-mediated oxidative stress in AECIIs and pulmonary fibrosis. Sohlh2 overexpression and the control A549 cells were pretreated with or without NAC (5 mM), then treated with 300 μM PA for 48 h. **a** Representative photographs of DHE fluorescent imaging of A549 cells. Scale bars: 50 μm. **b** The ROS levels in the indicated A549 cells were detected by FACS. **c** Representative images and quantification analysis of TUNEL staining in A549 cells. (*n* = 3). Scale bars: 20 μm. **d** qPCR analysis of proinflammatory cytokines mRNA levels in the indicated A549 cells. (*n* = 3). **e** ELISA analysis of IL-6, TNF-α, and TGF-β1 in the supernatant of A549 cells obtained from different groups. **f** Representative images showing gross lung morphology of mice obtained from the Control/HFD, Sohlh2 KI/HFD, Control/HFD + NAC, and Sohlh2 KI/HFD + NAC groups. (*n* = 5). **g** Representative images and quantification analysis of HE and Masson staining in sections from the Control and Sohlh2 KI lungs after 8-week HFD and NAC treatment. Scale bars: 100 μm. **h** Representative images of DHE fluorescent imaging of lung tissue sections in four groups. Scale bars: 100 μm; Representative images showing α-SMA (red) and nuclei (blue) in lung sections. Scale bars: 50 μm. **i**, **j** qPCR and Western blot analysis of profibrotic gene expression at mRNA and protein levels from indicated lung tissues. **k** qPCR analysis of proinflammatory cytokines mRNA levels from indicated lung tissues. Data are presented as the mean ± SD. ns. *P* > 0.05, *P < 0.05, **P < 0.01, and ***P < 0.001.

To further explore whether Sohlh2 accelerates the development of pulmonary fibrosis by regulating oxidative stress in AECIIs, A549 cells were pretreated with or without NAC, an inhibitor of oxidative stress, and then treated with PA for 48 h. The results of DHE staining and FACS analysis showed that NAC could partially block the production of ROS induced by Sohlh2 overexpression in A549 cells (Fig. 5a, b). Then we detected the cell apoptosis and inflammatory responses of PA-induced A549 cells pretreated with or without NAC. The data showed that NAC could significantly reduce the cell apoptosis (Fig. 5c) and the expression of IL-6, IL-1β, and TNF-α (Fig. 5d, e) in Sohlh2 overexpression A549 cells. These results suggest that NAC significantly alleviates oxidative stress, cell apoptosis, and inflammatory injury in Sohlh2 overexpression A549 cells after PA treatment.

The Control and Sohlh2 KI mice were fed with HFD, meanwhile treated with or without 100 μL NAC (30 mg/mL) for 8 weeks to evaluate whether scavenging ROS attenuates the occurrence and progression of pulmonary fibrosis. There were no obvious pathological changes in the lungs of the Sohlh2 KI mice after NAC treatment (Fig. 5f). HE and Masson staining of lung sections showed that NAC reduced the infiltration of inflammatory cells and the deposition of collagenous fibers in the Sohlh2 KI mice fed with HFD, and significantly improved the degree of fibrosis (Fig. 5g). As shown in Fig. 5h, Sohlh2 significantly boosted DHE and α-SMA fluorescent intensity in HFD-induced lung tissue sections, which was substantially attenuated by NAC treatment. Furthermore, the expression of fibrogenesis-related genes (Collagen I, FN, and α-SMA) was upregulated in the Sohlh2 KI/HFD group compared with the Control/HFD group, and this increase was partially reversed by the addition of NAC (Fig. 5I, j). To determine the protective effect of scavenging ROS on inflammation response, markers related to these processes in the serum or lung tissues of the Control/HFD group, Sohlh2 KI/HFD group, Control/HFD + NAC group, and Sohlh2 KI/HFD + NAC group were measured. Compared with the Sohlh2 KI/HFD group, NAC treatment markedly reduced the levels of IL-1β, IL-6, and TNF-α in the Sohlh2 KI mice (Fig. 5k). Taken together, these results suggest that NAC reduces the oxidative stress of AECIIs and pulmonary fibrosis caused by Sohlh2.

### Sohlh2 inhibits the activation of the p62/Keap1/Nrf2 signaling pathway by repressing p62 transcription in the lungs and AECIIs

**Fig. 6:**
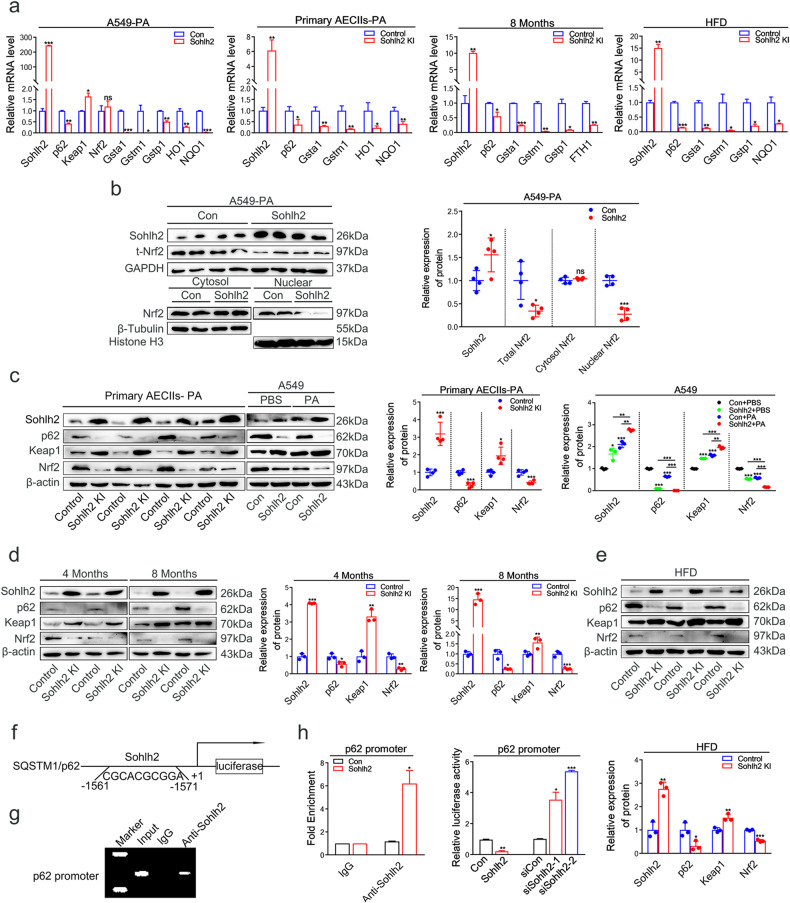
Sohlh2 inhibits the activation of the p62/Keap1/Nrf2 signaling pathway by repressing p62 transcription in the lungs and AECIIs. **a** qPCR analysis of Sohlh2, p62, Keap1, Nrf2 and its target genes in murine primary AECIIs and human A549 cells treated by 300 μM PA; qPCR analysis of Sohlh2, p62, Nrf2, and its downstream target genes expression in the indicated lungs of 8 M and HFD-fed mice. **b** Representative Western blot and quantification analysis showing the expression levels of Sohlh2, total Nrf2, and nuclear Nrf2 in Sohlh2 overexpressing A549 cells treated by PA. *n* = 4. GAPDH or β-Tubulin was used as a loading control for total or cytosolic proteins, and Histone H3 was used as a loading control for nuclear proteins. **c** Representative Western blot and quantification analysis showing the protein expression levels of Sohlh2, p62, Keap1, and total Nrf2 in mouse primary AECIIs and A549 cells treated by PA. **d**, **e** Representative Western blot and quantification analysis showing the expression levels of Sohlh2, p62, Keap1, and total Nrf2 in the Control and Sohlh2 KI lungs of 4 M, 8 M, and HFD-fed mice. **f** Predictive binding sites of Sohlh2 to p62 promoter region. **g** ChIP analysis of forced Sohlh2 expression and A549 cells using an anti-Sohlh2 antibody for p62 promoter. **h** p62 promoter-luciferase reporter activity in Sohlh2 overexpression and Sohlh2 shRNA A549 cells. Data are presented as the mean ± SD. ns. *P* > 0.05, *P < 0.05, **P < 0.01, and ***P < 0.001.

To dissect potential mechanisms underlying the Sohlh2-induced oxidative stress of cultured AECIIs in vitro, we performed qPCR and Western blot analysis to investigate the regulation of Sohlh2 on the Nrf2 signaling pathway [29], a classic intracellular antioxidant pathway, in mouse primary AECIIs and human A549 cells. qPCR results showed that mRNA levels of Nrf2 downstream target genes (Gsta1, Gstm1, Gstp1, HO1, NQO1, and FTH1) in Sohlh2-overexpression AECIIs were significantly decreased compared to the control group, however, mRNA levels of Nrf2 were unchanged between the two groups (Fig. 6a, Extended Data Fig. 2a). Considering that Nrf2 activates its target gene expression as a transcriptional factor only in the nucleus [30, 31], we next tried to determine the nuclear translocation of Nrf2 in AECIIs. As expected, we did observe that Nrf2 contents in the nuclear fraction were significantly lower in Sohlh2-overexpression AECIIs than in the control group, whereas cytosolic Nrf2 levels were similar between the two groups, but total protein levels of Nrf2 were greatly decreased in Sohlh2-overexpression AECIIs, compared to the control group (Fig. 6b); in contrast, Sohlh2 knockdown had the opposite effects (Extended Data Fig. 2b). Hence, these results suggest that elevation of Sohlh2 could suppress Nrf2 activity and its translocation but not its mRNA levels in AECIIs.

SQSTM1/p62 is an essential selective autophagy junction protein, which contains the ubiquitin-related domain to bind to Keap1. Keap1 degradation is induced by p62-mediated autophagy, resulting in the release and translocation of Nrf2 to the nucleus where it activates the transcription of antioxidant genes [11, 12, 32]. We thus determined protein levels of Keap1, p62, and total Nrf2 in mouse primary AECIIs and A549 cells. Western blot analysis revealed that Keap1 protein levels were significantly escalated, whereas the levels of p62 and total Nrf2 were dramatically diminished in mouse primary AECIIs and human A549 cells treated with PA, compared to the control group (Fig. 6c). In contrast, Sohlh2 knockdown A549 cells showed lower levels of Keap1 and higher levels of p62 and total Nrf2 in comparison to the controls (Extended Data Fig. 2c). The qPCR analysis showed that the mRNA level of p62 was greatly decreased in Sohlh2 overexpression primary AECIIs and human A549 cells (Fig. 6a). To further confirm the mechanism of Sohlh2 in the regulation of AECII oxidative stress during the development of pulmonary fibrosis, we examined the effect of Sohlh2 on regulating the p62/Keap1/Nrf2 signaling pathway in the lung tissues of 8 M and HFD-fed mice. Consistent with the results of in vitro experiments, mRNA levels of p62 and Nrf2 target genes (Gsta1, Gstm1, Gstp1, NQO1, and FTH1) in the lung tissues of 8 M and HFD-fed Sohlh2 KI mice were significantly downregulated compared to the Control mice (Fig. 6a). Western blot results showed that protein levels of p62 and total Nrf2 were significantly decreased, and Keap1 expression was markedly upregulated in the lung tissues of the Sohlh2 KI mice (Fig. 6d, e). Collectively, these data demonstrated a possible connection between Sohlh2 and the p62/Keap1/Nrf2 axis.

Sohlh2 is a member of the bHLH transcription factor family, which can bind to the conserved E-box sequence of the target gene promoters and regulate the expression of downstream target genes [22]. Therefore, we analyzed the binding site of Sohlh2 in the promoter region of p62 using the JASPAR database (Fig. 6f). To determine whether Sohlh2 could bind directly to the promoter region of p62 and represses its transcription, we performed a ChIP assay using Sohlh2-overexpression A549 cells and the control cells. ChIP analysis demonstrated the enrichment of Sohlh2 at the promoter of p62 (Fig. 6g). The results of the dual-luciferase reporter assay revealed that Sohlh2 overexpression reduced the luciferase activities driven by the p62 promoter. In comparison, Sohlh2 knockdown significantly enhanced the luciferase activities driven by the p62 promoter (Fig. 6h). These results indicated that Sohlh2 could directly bind to the promoter region of p62 to repress p62 transcription. Our study demonstrated that Sohlh2 overexpression inhibited the activation of the p62/Keap1/Nrf2 signaling pathway by repressing p62 transcription to induce oxidative stress in AECIIs, which led to severe pulmonary fibrosis.

### p62 overexpression prevents AECIIs from Sohlh2-induced oxidative stress damage and blocks the effect of Sohlh2 in activating the Keap1/Nrf2 signaling pathway

**Fig. 7:**
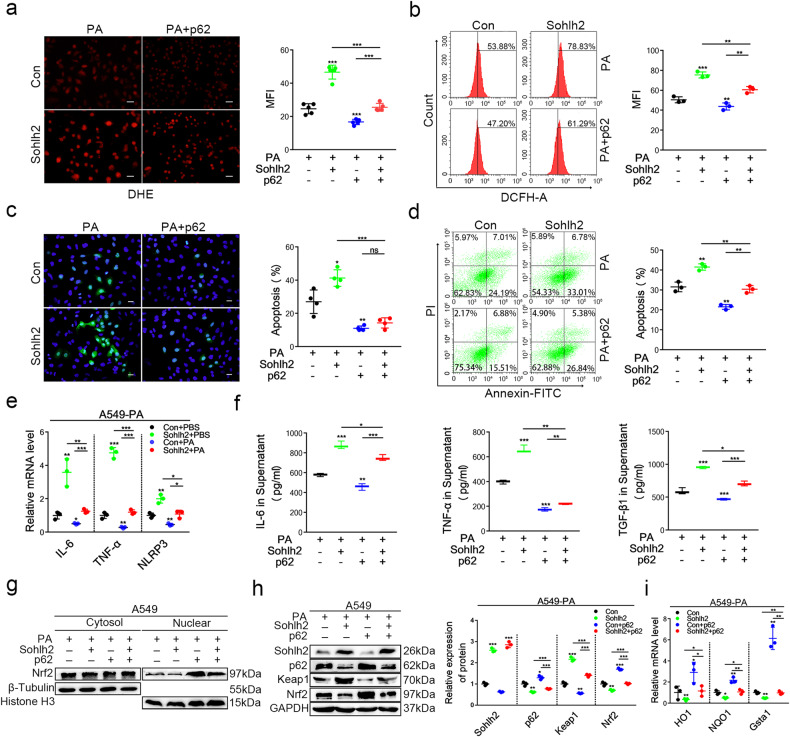
p62 overexpression prevents AECIIs from Sohlh2-induced oxidative stress damage and blocks the effect of Sohlh2 on the Keap1/Nrf2 signaling pathway. p62 overexpression or control plasmid was transfected into the control and Sohlh2 overexpressing A549 cells. **a** ROS levels were measured by DHE fluorescent intensity in four group A549 cells treated by PA. Scale bars: 50 μm. **b** Detection of ROS levels in four group A549 cells treated by PA by FACS. **c** Representative images and quantification analysis of TUNEL staining in four group A549 cells treated by PA. (*n* = 3). Scale bars: 20 μm. **d** Percentages of Annexin V-positive cells upon PA treatment were examined by FACS. **e**, **f** qPCR and ELISA analysis of the expression of proinflammatory cytokines from four group A549 cells treated by PA. **g** Representative Western blot images and quantification analysis showing the expression levels of Sohlh2, total Nrf2, and nuclear Nrf2 in the indicated A549 cells. **h** Representative Western blot images and quantification analysis showing the expression levels of Sohlh2, p62, Keap1, and total Nrf2 in the indicated A549 cells. **i** qPCR analysis of the expression of Nrf2 target genes (HO1, NQO1, Gsta1) in the indicated A549 cells. Data are presented as the mean ± SD. *P < 0.05, **P < 0.01, and ***P < 0.001.

To confirm whether p62 mediates Sohlh2-induced oxidative stress damage in AECIIs, the p62-overexpression plasmid was transfected into Sohlh2 overexpressing AECIIs. The results of DHE staining and FACS analysis showed that the overexpression of p62 could partially attenuate the production of ROS in Sohlh2 overexpression AECIIs (Fig. 7a, b). We detected the cell apoptosis and inflammatory responses of Sohlh2 overexpressing A549 cells transfected with or without p62 plasmid, the data showed that p62 could markedly reduce the cell apoptosis and the expression of IL-1β, IL-6, and TNF-α in Sohlh2 overexpression A549 cells (Fig. 7c–f). To clarify whether p62 mediates the effect of Sohlh2 on activating the Keap1/Nrf2 signaling pathway, the expression of Keap1, total Nrf2, and its target genes were examined in Sohlh2 overexpression A549 cells transfected with p62 overexpression plasmid. Western blot results confirmed that p62 partially reduces Sohlh2-mediated Nrf2 entry into the nucleus and activation of the Keap1/Nrf2 signaling pathway (Fig. 7g–i).

**Fig. 8:**
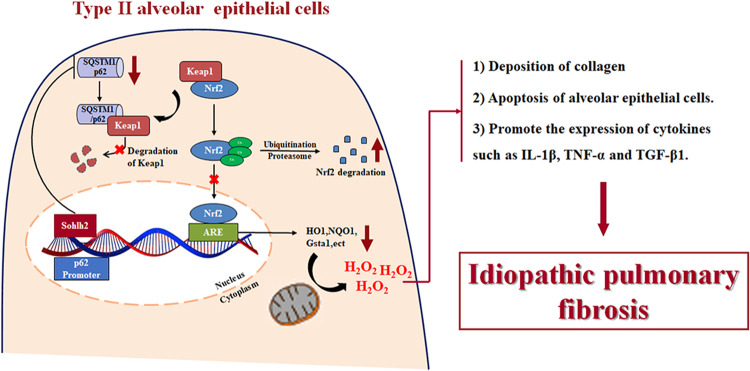
Sohlh2 promotes pulmonary fibrosis via suppressing the activation of the p62/Keap1/Nrf2 signaling pathway and aggravating oxidative stress of AECIIs. In the occurrence and progression of pulmonary fibrosis, Sohlh2 can downregulate the transcriptional activity of p62 by directly binding to the promoter region of p62, activating the Keap1/Nrf2 signaling pathway to result in the production of ROS in AECIIs, leading to inflammation, cells apoptosis, and fibrosis of lung tissues under different conditions. Therefore, targeted Sohlh2 may prevent age-related and stress-induced pulmonary fibrosis and provide a new way for clinical treatment of IPF with anti-oxidation.

Indeed, the qPCR analysis showed that p62 partially blocked the expression of Nrf2 target genes caused by Sohlh2 overexpression in AECIIs. These results reveal that p62 mediates the effects of Sohlh2 on oxidative stress damage and activation of the Keap1/Nrf2 signaling pathway in AECIIs (Fig. 8).

## Discussion

Here we uncovered a critical role for Sohlh2 in age-related and HFD-induced pulmonary fibrosis. Overexpression of Sohlh2 in AECIIs led to mitochondrial dysfunction, excessive ROS production, and fibrogenesis in the murine lungs.

Alveolar epithelial cells are composed of AECIs and AECIIs. In addition to the secretion of lung surfactant, AECIIs function as the main progenitors of alveoli, which differentiate into AECIs for alveolar repair [5]. Accumulating evidence has demonstrated that impaired AECIIs function caused by aging and environmental stress is a major contributor to pulmonary fibrogenesis [33]. During the development of pulmonary fibrosis, mitochondrial dysfunction is a key pathogenic event in AECIIs injury [8, 34]. The depletion of the mitochondrial fusion proteins mitofusin1 (Mfn1) and mitofusin2 (Mfn2) in murine AECIIs leads to impaired lipid metabolism and spontaneous lung fibrosis [35]. Using AECII specific Sohlh2 CKI mouse model, we confirmed that Sohlh2 overexpression caused mitochondrial damage in AECIIs and spontaneous lung fibrosis, and Sohlh2 in AECIIs also largely enhanced HFD-driven oxidative stress, fibrotic remodeling, inflammation, and cell death. Our findings provided evidence that Sohlh2 may be a therapeutic target in lung fibrosis. Our current work also supports the present theory that injury to AECIIs encourages the aberrant alveolar repair process eventually leading to the extensive lung remodeling observed in IPF.

Oxidative stress plays a very important role in age-related lung fibrosis [36, 37], which is characterized by the accumulation of MDA and ROS. ROS act as a double-edged sword in tissues, where low levels of ROS are beneficial but excessive accumulation leads to inflammation and fibrogenesis. The high levels of ROS are associated with the damage of epithelial cells in IPF [2, 3, 38]. HFD contributes to mitochondrial alternation and ROS generation, which triggers the tendency of pulmonary fibrogenesis. Sohlh2 overexpression led to age-related pulmonary fibrosis and augmented HFD-induced pulmonary fibrosis via induction of ROS generation. ROS inhibitor (NAC) blocked the effects of Sohlh2 on redox imbalance and fibrogenesis in the lungs. These findings confirmed that Sohlh2 induced spontaneous pulmonary fibrogenesis via augmenting ROS production for the first time.

The endogenous ROS in cells is mainly from mitochondrial oxidative phosphorylation and activation of NADPH oxidases (NOXs). Targeting NOX4 promotes the regression of pulmonary fibrosis [39, 40]. We first detected the expression of NADPH oxidases at mRNA and protein levels. None of them was upregulated by Sohlh2 (Extended. Fig. 3a, b). Elevated levels of ROS are counteracted by the antioxidant defense system [41]. Nrf2, a key redox balance regulator, maintains the phenotype of AECIIs and enhances the defense of alveolar cells against inflammatory damage [42]. Loss of Nrf2 leads to increased ROS production and exacerbates fibrosis [14]. Our results showed that Sohlh2 dramatically downregulated Nrf2 at the protein level, while not at the mRNA level. p62 facilitates the competitive binding of Keap1 and prolongs the half-life of Nrf2 [12, 43]. The results of the ChIP and luciferase reporter showed that p62 was the direct target of Sohlh2. Sohlh2 inhibited the activation of Nrf2 signaling via repressing p62 transcription. p62 mediated the effects of Sohlh2 on ROS production and the development of pulmonary fibrosis. Together, our findings indicate that Sohlh2 attenuates p62/Keap1/Nrf2 signaling pathway, and triggers increased ROS production, driving fibrogenesis in the lung. There are several E-box sequences located in the promoter of p62. In the future study, it is worth exploring which E-box sequence mediates the inhibitory effect of Sohlh2 on p62 transcription.

In conclusion, our findings defined a novel role of Sohlh2 in age-related and stress-induced fibrogenesis. Our results showed that the Sohlh2/p62/Keap1/Nrf2 axis functioned in the regulation of ROS generation, thereby governing inflammation, cell apoptosis, and fibrogenesis in the lung under different conditions. Our study provided proof that targeting Sohlh2 may prevent age-related and stress-induced pulmonary fibrosis.

### Supplementary information

Extended Data Figures and Extended Data Tables

Original western blots

aj-checklist

## Data Availability

The related data during the present study are available from the corresponding author on reasonable request.
