# Supplementary material for: Sohlh2 promotes pulmonary fibrosis via repression of p62/Keap1/Nrf2 mediated anti-oxidative signaling pathway
Source: Cell Death Dis. 2023 Oct 24;14(10):698. doi: 10.1038/s41419-023-06179-z (PMC10598036; doi:10.1038/s41419-023-06179-z)
Supplement: Supplementary file 1 — Extended Data Figures and Extended Data Tables [file 41419_2023_6179_MOESM1_ESM.docx]

**Sohlh2 promotes pulmonary fibrosis via repression of p62/keap1/Nrf2 mediated anti-oxidative signaling pathway**

Lanlan Liu^1^, Xiaoli Zhang^1^, Ruihong Zhang^1^, LiyanWang^2^, Sujuan Zhi^1^, Xiaoning Feng^1^, Xuyue Liu^1^, Ying Shen^1^, Jing Hao ^1*^


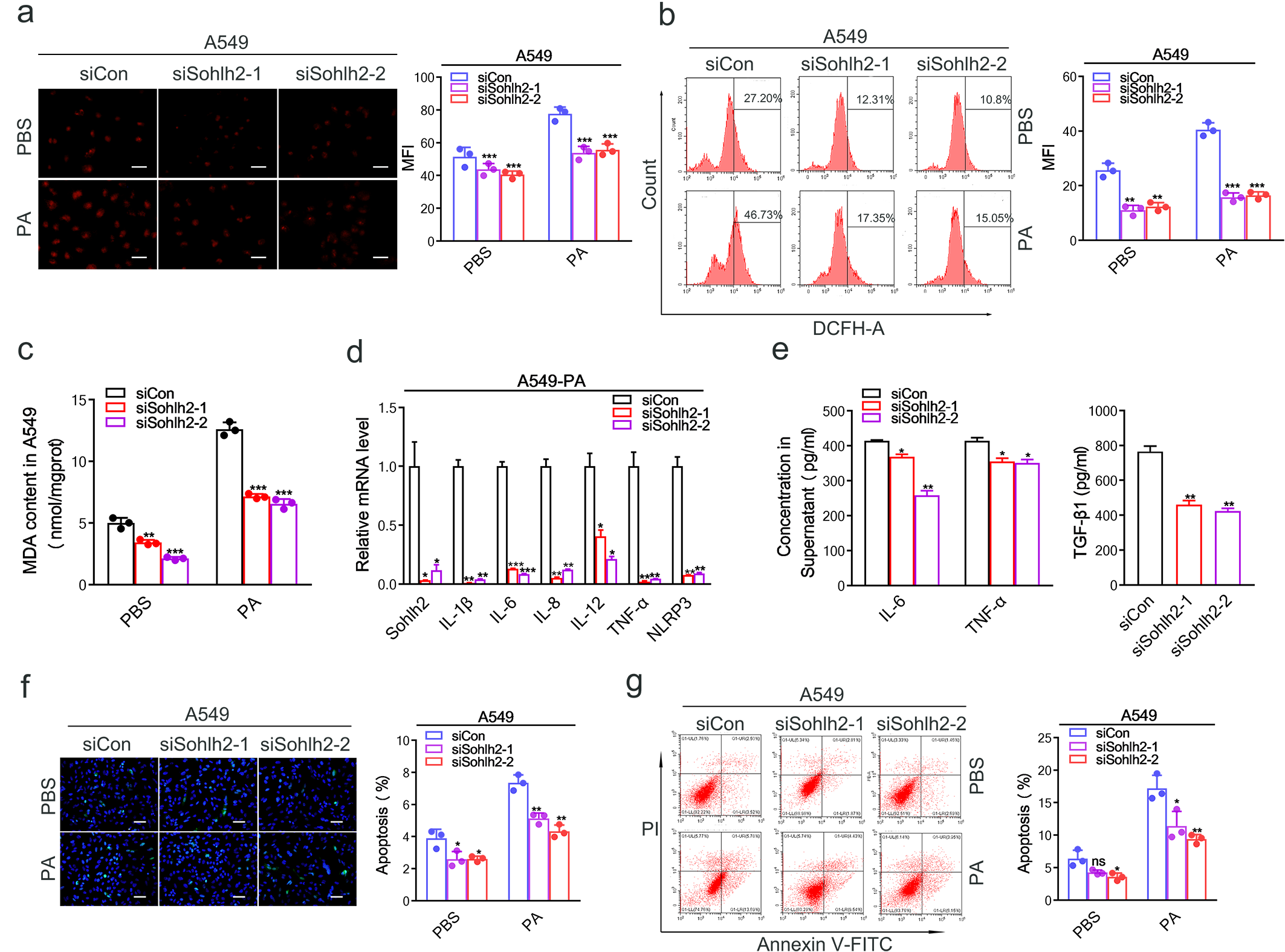


[**Extended Data Fig. 1**](https://www.nature.com/articles/s41586-022-05380-y/figures/5) **Sohlh2 enhances oxidative stress in cultured AECIIs.** (a) ROS levels, measured by DHE fluorescent intensity in Sohlh2 knockdown A549 cells treated with or without 300μM PA. Scale bars: 50μm. (b) Detection of ROS level and average immunofluorescence intensity of Sohlh2 knockdown A549 cells treated with or without 300μM PA by FACS. (c) The levels of MDA were shown in Sohlh2 knockdown A549 cells treated by 300μM PA. (d) qPCR analysis of proinflammatory cytokines mRNA levels from Sohlh2 knockdown A549 cells treated by 300μM PA. (n=3). (e) ELISA analysis of IL-6, TNF-α, and TGF-β1 in the culture medium of Sohlh2 knockdown A549 cells treated by 300μM PA. (f) Representative images and quantification analysis of TUNEL staining in Sohlh2 knockdown A549 cells treated with or without 300μM PA. (n=3). Scale bars: 20μm. (g) Percentages of Annexin V-positive cells upon 300μM PA treatment were examined by FACS. Cells treated with PBS served as the control. (n=3). Data are presented as the mean ± SD. ns. *P* > 0.05, **P* < 0.05, ***P* < 0.01, and ****P* < 0.001.


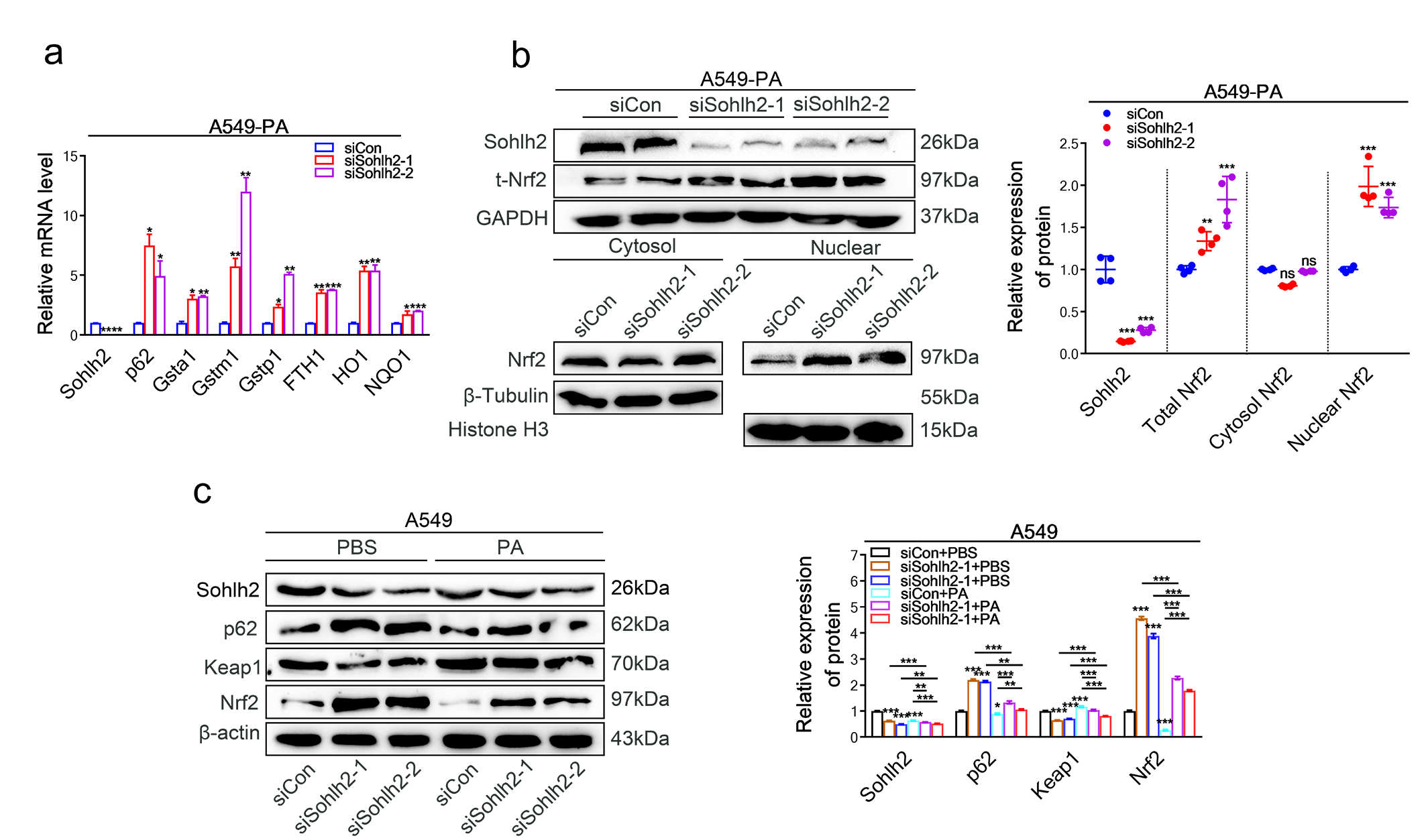


**Extended Data Fig. 2 Sohlh2 inhibits the activation of the p62/Keap1/Nrf2 signaling pathway in AECIIs.** (a) qPCR analysis of Sohlh2, p62, and Nrf2 target genes in Sohlh2 knockdown A549 cells treated by 300μM PA. (b) Representative Western blot and quantification analysis showing the expression levels of Sohlh2, total Nrf2, and nuclear Nrf2 in Sohlh2 knockdown A549 cells treated by 300μM PA. n=4. GAPDH or β-Tubulin was used as a loading control for total or cytosolic proteins, and Histone H3 was used as a loading control for nuclear proteins. (c) Representative Western blot and quantification analysis showing the protein expression levels of Sohlh2, p62, Keap1, and total Nrf2 in Sohlh2 knockdown A549 cells treated by 300μM PA. Cells treated with PBS served as the control. Data are presented as the mean ± SD. ns. *P* > 0.05, **P* < 0.05, ***P* < 0.01, and ****P* < 0.001.


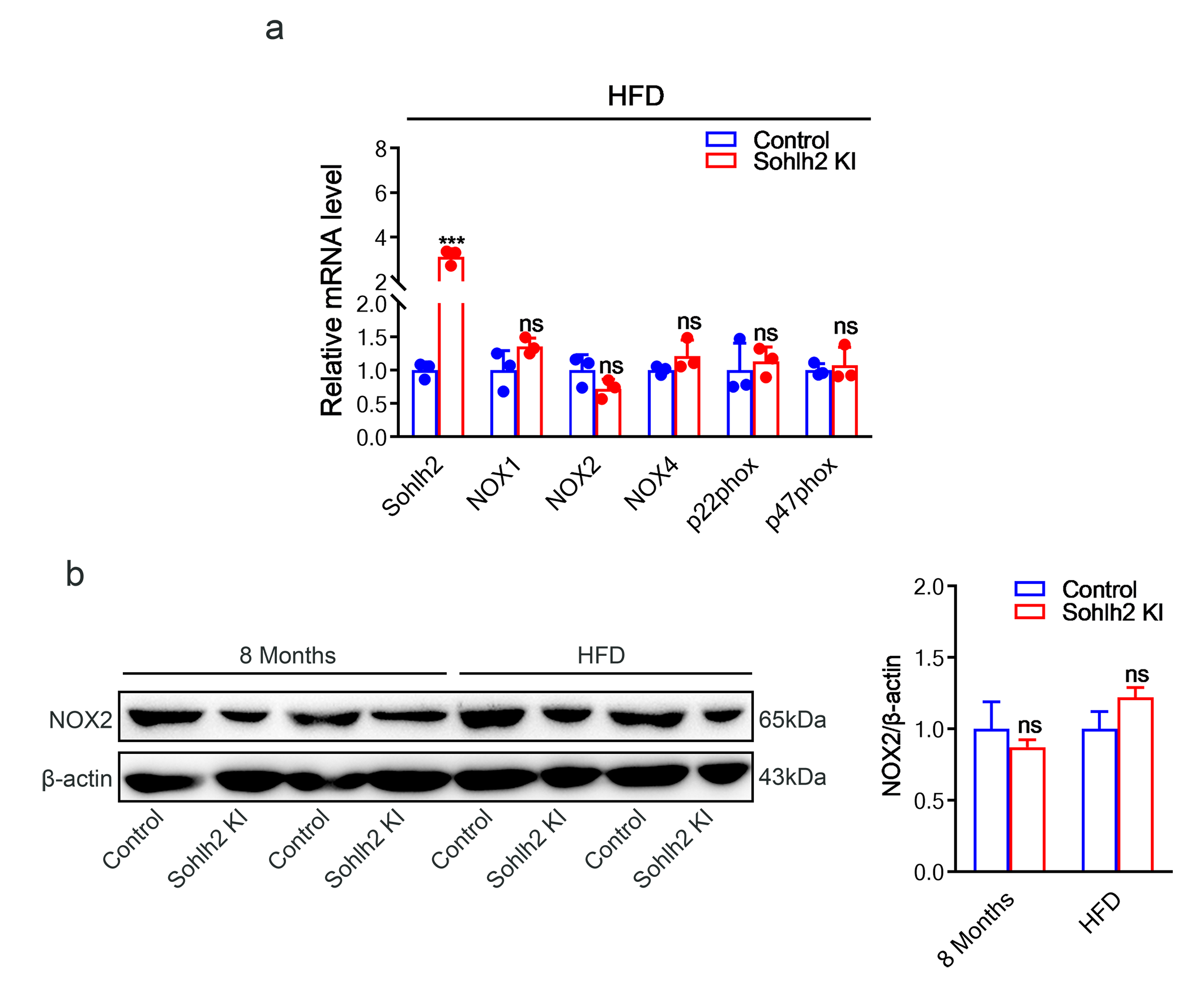


**Extended Data Fig. 3 The expression of NOX1, NOX2, NOX4, p22phox, and p47phox at mRNA and protein levels in the lungs of Sohlh2 KI mice**. (a) qPCR analysis of Sohlh2, NOX1, NOX2, NOX4, p22phox, and p47phox in the lungs of the Sohlh2 KI mice. (b) Representative Western blot and quantitative data of NOX2 protein expression in the 8M and HFD-fed Sohlh2 KI mice. Data are presented as the mean ± SD. ns. *P* > 0.05 and ****P* < 0.001.

**Extended Data Table 1**. Primer sequences for PCR

| **Gene name** |  | **Primer sequences（5’ → 3’）** |
| --- | --- | --- |
| *Sftpc*^CreERT2+^ | Forward 1 | 5’ - TGCTTCACAGGGTCGGTAG - 3’ |
|  | Reverse 1 | 5’ - ACACCGGCCTTATTCCAAG - 3’ |
|  | Reverse 2 | 5’ - CATTACCTGGGGTAGGACCA - 3’ |
| *Sohlh2-Loxp* | *Sohlh2-Loxp-F* | 5’-CAGACTTGTGGGATACAGAAGAC-3’ |
|  | *Sohlh2-Loxp-R* | 5’-CTGCTGTCCATTCCTTATTCCATAG-3’ |
|  | *Sohlh2-Loxp-WT* | 5’-AGTCCACCTCACTCCTCATAAC-3’ |

**Extended Data Table 2**. Primer sequences for quantitative RT-PCR

| **Gene name** | **Primer sequences（5’ → 3’）** | |
| --- | --- | --- |
|  | **Human** | **Mouse** |
| GAPDH-F | GAAAGCCTGCCGGTGACTAA | AGGAGAGTGTTTCCTCGTCC |
| GAPDH-R | AGGAAAAGCATCACCCGGAG | TGAGGTCAATGAAGGGGTCG |
| Sohlh2-F | TTGTTTGCAGTAGAACAGGCTC | TTATGTGAAGCAGGTCCGGG |
| Sohlh2-R | ATTGTGGCAGCCAAGACTGA | TGGAAGGAACAGCTCGATGG |
| IL-1β-F | CCTGAGCTCGCCAGTGAAAT | GCCACCTTTTGACAGTGATGAG |
| IL-1β-R | GTCGGAGATTCGTAGCTGGA | ATGTGCTGCTGCGAGATTTG |
| IL-6-F | CCAGAGCTGTGCAGATGAGT | TGGTCTTCTGGAGTACCATAGC |
| IL-6-R | GCATTTGTGGTTGGGTCAGG | GTGACTCCAGCTTATCTCTTGG |
| IL-8-F | AAGTTTTGAAGAGGGCTGAGA | TGTTCACAGGTGACTGCTCC |
| IL-8-R | TGCTTGAAGTTTCACTGGCAT | AGCCCATAGTGGAGTGGGAT |
| IL-12-F | CAGAAGGCCAGACAAACTCT | CATCTGGCGTCTACACTGCT |
| IL-12-R | GGTCTCTCTGGAATTTAGGCA | GGTAGCGTGATTGACACATGC |
| TNF-α-F | TCAGAGGGCCTGTACCTCAT | ACTGAACTTCGGGGTGATCG |
| TNF-α-R | GGAGGTTGACCTTGGTCTGG | CCACTTGGTGGTTTGTGAGTG |
| iNOS-F | CGCATGACCTTGGTGTTTGG | GAGCGCTCTAGTGAAGCAAAG |
| iNOS-R | CATAGACCTTGGGCTTGCCA | CTCACATACTGTGGACGGGT |
| NLRP3-F | GCTGGCATCTGGGGAAACCT | TACCCAAGGCTGCTATCTGGA |
| NLRP3-R | GTCCTTAGGCTTCGGTCCAC | TTGCAACGGACACTCGTCA |
| TGF-β1-F | TATTGAGCACCTTGGGCACTG | ACGTCACTGGAGTTGTACGG |
| TGF-β1-R | GCAGGAACTCCTCCCTTAACC | GGGGCTGATCCCGTTGATT |
| CTGF-F | AGGATGTGCATTCTCCAGCC | AGAACTGTGTACGGAGCGTG |
| CTGF-R | GCCACAAGCTGTCCAGTCTA | GTGCACCATCTTTGGCAGTG |
| Collagen I -F | GCAGCCCTGGTGAAAATGGA | AGCACGTCTGGTTTGGAGAG |
| Collagen I -R | CAGCACCAGTAGCACCATCA | GACATTAGGCGCAGGAAGGT |
| FN-F | TCAGCTTCCTGGCACTTCTG | GGCCACCATTACTGGTCTGG |
| FN-R | TCTTGTCCTACATTCGGCGG | GGAAGGGTAACCAGTTGGGG |

**Continued Extended Data** **Table 2**. Primer sequences for quantitative RT-PCR

| **Gene name** | **Primer sequences（5’ → 3’）** | |
| --- | --- | --- |
|  | **Human** | **Mouse** |
| α-SMA-F | GAGGGAAGGTCCTAACAGCC | CTACGAACTGCCTGACGGG |
| α-SMA-R | TAGTCCCGGGGATAGGCAAA | GCTGTTATAGGTGGTTTCGTGG |
| p62-F | TCAAGCAGTATCCCAAGGAGG | GATAGCCTTGGAGTCGGTGG |
| p62-R | TCACATGGGGGTCCAAAGAC | GATCAGCCTCTGTAGATGGGTC |
| Keap1-F | ACGGGACAAACCGCCTTAAT | CTCAACCGCTTGCTGTATGC |
| Keap1-R | ATACAGTTGTGCAGGACGCA | GTGTGATCATCCGCCACTCA |
| Nrf2-F | CTGCCAACTACTCCCAGGTT | TGAAGCTCAGCTCGCATTGA |
| Nrf2-R | CAAGTGACTGAAACGTAGCCG | TGCTCCAGCTCGACAATGTT |
| HO1-F | AGTCTTCGCCCCTGTCTACT | GAAATCATCCCTTGCACGCC |
| HO1-R | CTTCACATAGCGCTGCATGG | CCTGAGAGGTCACCCAGGTA |
| NQO1-F | GGCTGGTTTGAGCGAGTGT | TAGCCTGTAGCCAGCCCTAA |
| NQO1-R | ACCAGTGGTGATGGAAAGCAC | ACAATCAGGGCTCTTCTCGC |
| Gsta1-F | TGATCCTCCTTCTGCCCGTA | CCACCTACTGGAAGTTCTCCTC |
| Gsta1-R | CAAAGGCAGGGAAGTAGCGA | GAGGCTGCTGATTCTGCTCT |
| Gstm1-F | ATACTCTGAGCCCTGCTCG | GATCCGTGCAGACATTGTGG |
| Gstm1-R | GTCCCAGTACCCCAGTATCAT | TGGCTGTCACCACCTTTAGAC |
| Gstp1-F | ATACCATCCTGCGTCACCTG | AGCACTGAATCCGCACCCA |
| Gstp1-R | CCCGCCTCATAGTTGGTGTA | GCATTCGCATGGCCTCACA |
| FTH1-F | CCAGAACTACCACCAGGACTC | GGAGCATGCCGAGAAACTGA |
| FTH1-R | ACAGGTAAACGTAGGAGGCG | TCTCCCAGTCATCACGGTCT |

**Extended Data Table 3**. Antibodies

| **Antibody Name** | **Company** | **Art. NO.** |
| --- | --- | --- |
| Anti-Sohlh2 Antibody | NOVUS | NBP2-20453 |
| Anti-β-Tubulin Antibody | Affinity | AF7011 |
| Anti-Collagen I Antibody | CST | 95855S |
| Anti-FN Antibody | BOSTER | MA1116 |
| Anti-α-SMA Antibody | Affinity | AF1032 |
| Anti-TGF-β1 Antibody | Affinity | BF8012 |
| Anti-E-cadherin Antibody | Affinity | AF0131 |
| Anti-GAPDH Antibody | Affinity | AF7021 |
| Anti-SQSTM1/p62 Antibody | Affinity | AF5384 |
| Anti-Keap1 Antibody | Affinity | AF5622 |
| Anti-Nrf2 Antibody | Abmart | T55136 |
| Anti-β-actin Antibody | Affinity | AF7018 |
| Anti-Histone H3 Antibody | Affinity | AF0863 |
| Anti-SP-C Antibody | Affinity | DF6647 |
| Peroxidase AffiniPure Goat  Anti-Rabbit IgG (H+L) | Jackson ImmunoReasearch | 111-035-003 |
| Peroxidase AffiniPure Goat  Anti-Mouse IgG (H+L) | Jackson ImmunoReasearch | 115-405-166 |
| Dylight 594, Goat Anti-Rabbit lgG | Abbkine | A23420 |
