# Supplementary figures and images for: Sohlh2 promotes pulmonary fibrosis via repression of p62/Keap1/Nrf2 mediated anti-oxidative signaling pathway
Source: Cell Death Dis. 2023 Oct 24;14(10):698. doi: 10.1038/s41419-023-06179-z (PMC10598036; doi:10.1038/s41419-023-06179-z)

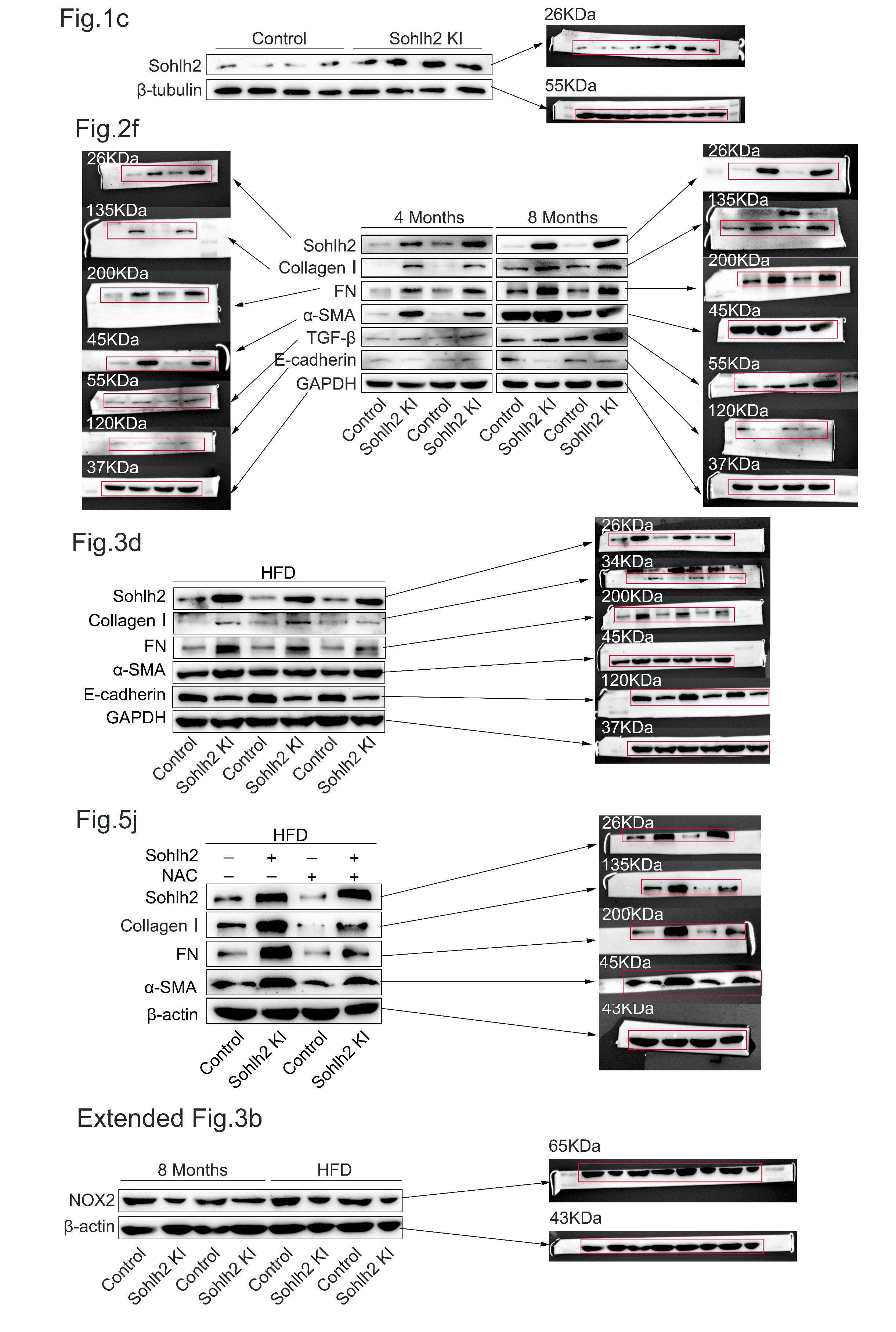


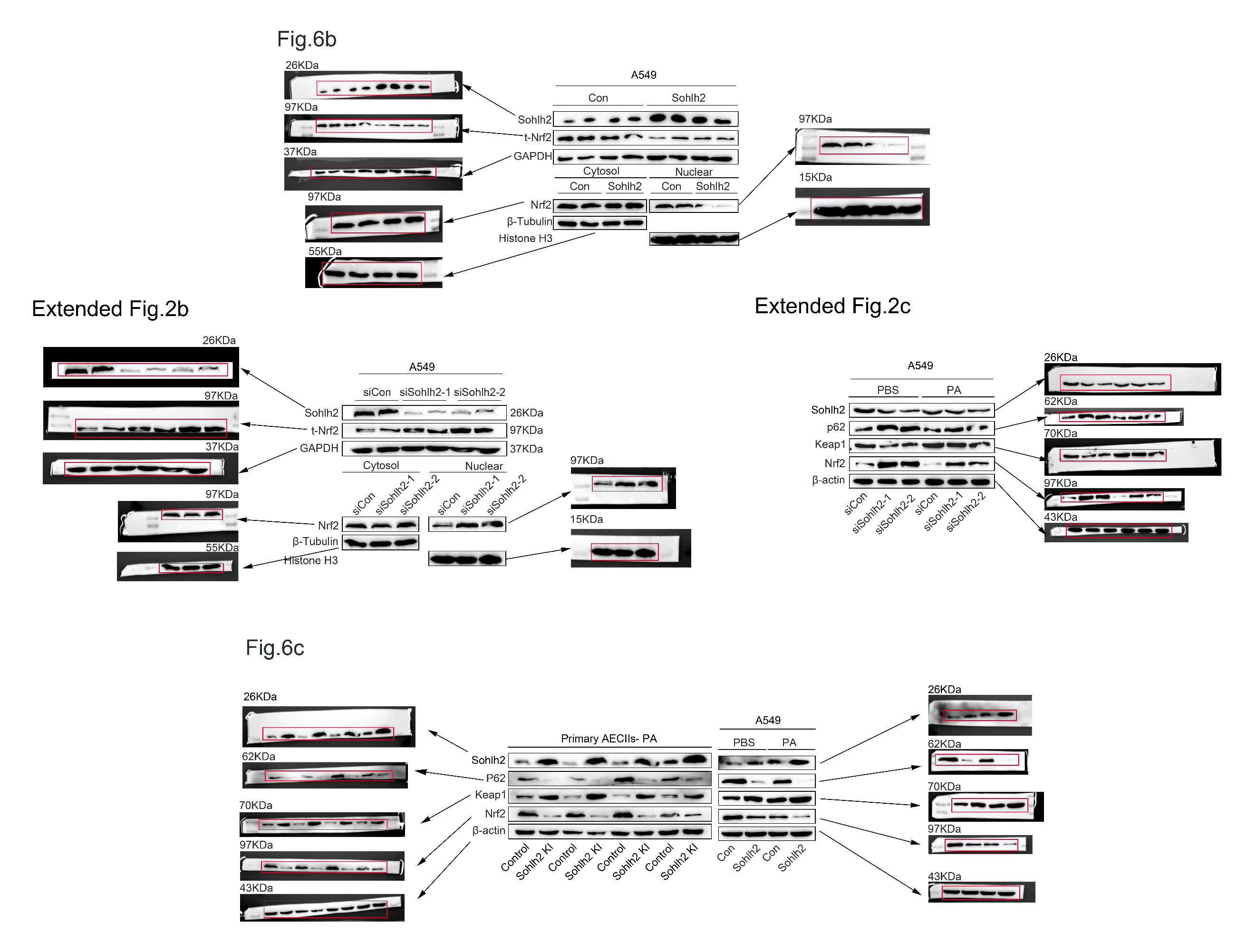

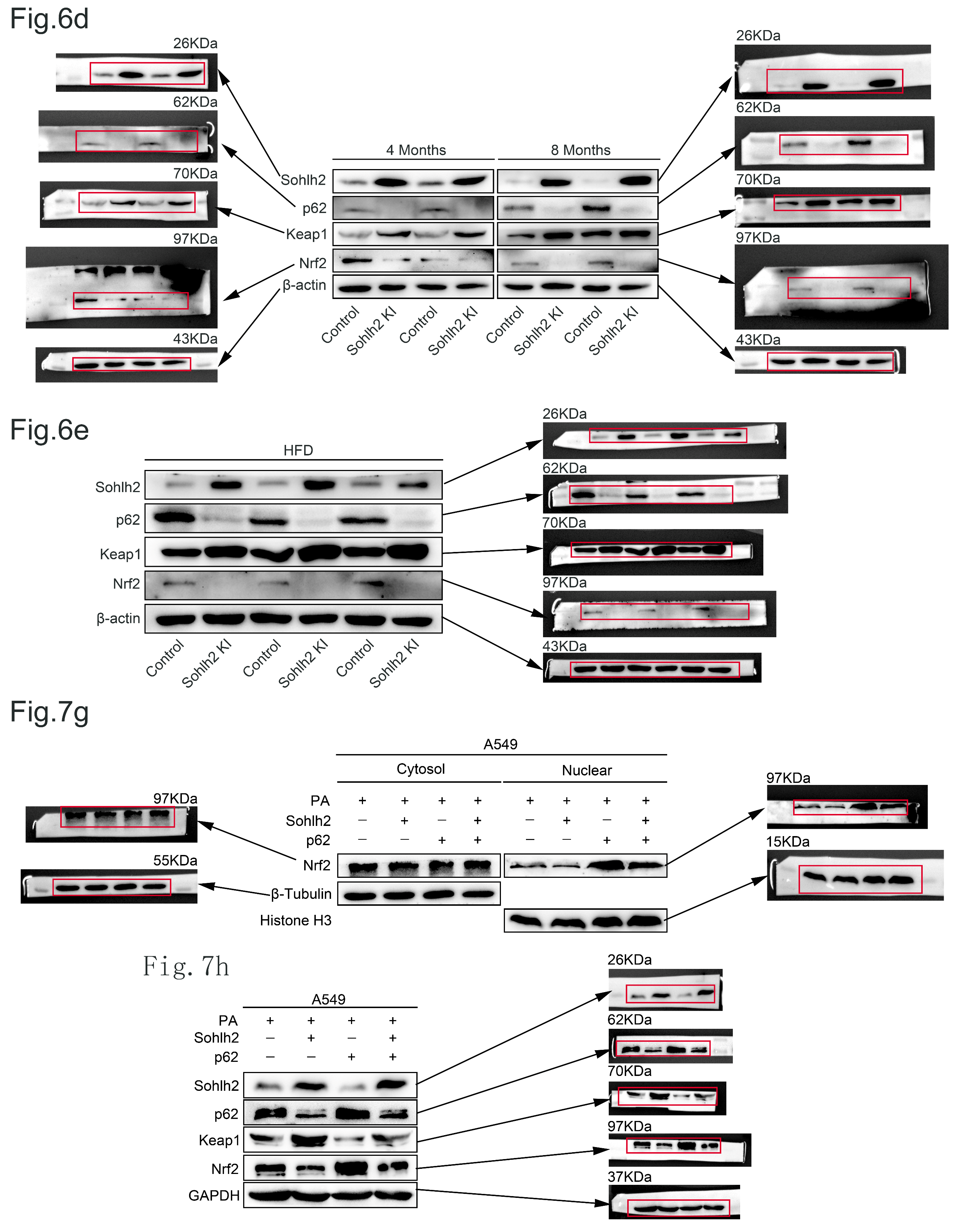

Supplement: Supplementary file 2 — Original western blots [file 41419_2023_6179_MOESM2_ESM.docx]
